# Arginine deiminase PEG20 inhibits growth of small cell lung cancers lacking expression of argininosuccinate synthetase

**DOI:** 10.1038/bjc.2011.524

**Published:** 2011-12-01

**Authors:** M P Kelly, A A Jungbluth, B-W Wu, J Bomalaski, L J Old, G Ritter

**Affiliations:** 1 Ludwig Institute for Cancer Research Ltd, New York Branch of Human Cancer Immunology at Memorial Sloan-Kettering Cancer Center, New York, NY, USA; 2 Polaris Group, 6370 Nancy Ridge Drive, Suite 106, San Diego, CA, USA

**Keywords:** arginine deiminase, SCLC, argininosuccinate synthetase, *in vivo*, autophagy

## Abstract

**Background:**

Some cancers have been shown to lack expression of argininosuccinate synthetase (ASS), an enzyme required for the synthesis of arginine and a possible biomarker of sensitivity to arginine deprivation. Arginine deiminase (ADI) is a microbial enzyme capable of efficiently depleting peripheral blood arginine.

**Methods:**

Argininosuccinate synthetase expression was assessed in human small cell lung cancer (SCLC) by immunohistochemistry (IHC), with expression also assessed in a panel of 10 human SCLC by qRT-PCR and western blot. Proliferation assays and analyses of apoptosis and autophagy assessed the effect of pegylated ADI (ADI-PEG20) *in vitro*. The *in vivo* efficacy of ADI-PEG20 was determined in mice bearing SCLC xenografts.

**Results:**

Approximately 45% of SCLC tumours and 50% of cell lines assessed were negative for ASS. Argininosuccinate synthetase-deficient SCLC cells demonstrated sensitivity to ADI-PEG20, which was associated with the induction of autophagy and caspase-independent cell death. Arginine deiminase-PEG20 treatment of ASS-negative SCLC xenografts caused significant, dose-dependent inhibition of tumour growth of both small and established tumours.

**Conclusion:**

These results suggest a role for ADI-PEG20 in the treatment of SCLC, and a clinical trial exploring this therapeutic approach in patients with ASS-negative SCLC by IHC has now been initiated.

Small cell lung cancer (SCLC) is characterized by a strong initial response to chemotherapy, although the majority of patients go on to relapse (Dowell, 2010; Rodriguez and Lilenbaum, 2010). In those patients for whom first-line chemotherapy fails, the chance of response to secondary treatments remains around 10%, and overall survival in these patients is only 3–4 months. Further, current treatment lacks tumour specificity and results in numerous toxicities, and may consequently limit the administration of therapeutics to below the maximally effective dose (Demedts *et al*, 2010; Dowell, 2010; Rodriguez and Lilenbaum, 2010). This situation highlights the need for the continued development of effective anti-cancer agents with high therapeutic indices.

Increased understanding of tumour biology has allowed for the identification of various cellular characteristics more associated with malignancy than normal tissues, and the subsequent development of therapeutics designed to exploit these differences. Although progression to a malignant phenotype may confer cancer cells with various proliferative advantages over normal cells, this process may also lead tumours to lose certain regular metabolic capabilities (Kroemer and Pouyssegur, 2008; Tennant *et al*, 2010). Indeed, certain tumours have been shown to lack expression of the enzyme argininosuccinate synthetase (ASS) required for the synthesis of arginine through the urea cycle, and therefore these tumours must rely on exogenous sources for protein synthesis requirements (Feun *et al*, 2008; Delage *et al*, 2010). Interestingly, loss of ASS expression has been observed as a biomarker for resistance to platinum-based chemotherapy in ovarian cancer and as a predictor of metastases in osteosarcoma, highlighting the complex role amino-acid metabolism has in the overall functioning of cells (Nicholson *et al*, 2009; Delage *et al*, 2010; Kobayashi *et al*, 2010). Thus, loss of ASS expression may be both prognostic and potentially predictive of response to arginine deprivation therapy.

Loss of ASS expression has been most widely associated with melanoma and hepatocellular carcinoma, although it has now been identified in other tumour types including pancreatic cancer, leukaemia, prostate cancer and renal cell carcinoma (Gong *et al*, 2000; Ensor *et al*, 2002; Yoon *et al*, 2007; Bowles *et al*, 2008; Kim *et al*, 2009b). Conversely, other tumour types such as colorectal cancer most often demonstrate robust expression of ASS (Shen and Shen, 2006; Shen *et al*, 2006). The loss of ASS in certain tumours is thought to be through epigenetic silencing through hypermethylation of the ASS gene promoter, although this has not been observed in all ASS-deficient cancers (Szlosarek *et al*, 2006; Nicholson *et al*, 2009). Poorly characterized post-translational silencing or recessive mutations may also be responsible for the loss of ASS expression observed in disparate tumour types.

The reliance of ASS-deficient cancers on exogenous arginine for continued growth initiated the development of therapeutics aiming to deprive such tumours of arginine through systemic catabolism. Arginine deiminase (ADI) is a bacterial enzyme originally isolated from various strains of Mycoplasma and degrades arginine by hydrolyzing it to its precursor citrulline (Delage *et al*, 2010). As the bacterial enzyme is highly immunogenic in humans, therapeutic preparations of ADI have been conjugated with polyethylene glycol (20 000 Da; ADI-PEG20) that serves to reduce the immunogenicity of the enzyme while greatly improving its pharmacokinetic half-life in serum (Feun and Savaraj, 2006; Feun *et al*, 2008; Ni *et al*, 2008). The precedent for a catabolic therapeutic enzyme targeting a specific amino acid is PEG-asparaginase (Oncaspar), which has been used successfully in the treatment of childhood acute lymphoblastic leukaemia due to its asparagine auxotrophy arising from a loss of the enzyme asparagine synthetase (Feun and Savaraj, 2006).

As it was first apparent that many melanomas are auxotrophic for arginine, the majority of studies assessing ADI-PEG20 and other arginine catabolizing enzymes have been undertaken in this tumour type (Feun and Savaraj, 2006; Shen and Shen, 2006; Ni *et al*, 2008). Treatment of ASS-deficient melanoma and hepatocellular carcinomas with ADI-PEG20 has been observed to mediate inhibition of growth *in vitro* and the retardation of tumour growth in xenografts (Sugimura *et al*, 1992; Takaku *et al*, 1992; Ensor *et al*, 2002). Clinical trials of ADI-PEG20 have followed in both melanoma and hepatocellular carcinoma (Izzo *et al*, 2004; Ascierto *et al*, 2005; Delman *et al*, 2005; Ott *et al*, 2009; Feun *et al*, 2010; Glazer *et al*, 2010; Yang *et al*, 2010). Pharmacodynamic results from these studies demonstrated that a dose of 160 IU m^−2^ ADI-PEG20 was sufficient to reduce plasma arginine levels from ∼130 *μ*M to below the level of detection (<2 *μ*M). Further, these trials provided some indication of clinical response to arginine deprivation therapy, with ADI-PEG20 achieving response rates of 25% and 47% in melanoma and hepatocellular carcinoma, respectively (Izzo *et al*, 2004; Ascierto *et al*, 2005; Delman *et al*, 2005). However, similar rates of sustained clinical responses have not been observed in further larger studies of ADI-PEG20 in metastatic melanoma or hepatocellular carcinoma, although an apparent increase in overall survival has been observed (Feun *et al*, 2010; Glazer *et al*, 2010; Yang *et al*, 2010). The current study is the first to examine the extent of arginine auxotrophy in SCLC and to explore the effectiveness of ADI-PEG20 arginine deprivation therapy in this disease.

## Materials And Methods

### Cell lines, antibodies and chemicals

A panel of 10 SCLC was obtained from the cell bank of the Ludwig Institute for Cancer Research, New York Branch at Memorial Sloan-Kettering Cancer Center (MSKCC). Cells were either established at MSKCC or purchased from the American Type Culture Collection (ATCC; Manassas, VA, USA). Cells were grown in RPMI-1640 media supplemented with 10% v/v fetal calf serum, 5% w/v penicillin/streptomycin (penicillin G 5000 units ml^−1^ per streptomycin sulphate 5000 mg ml^−1^ and 1% L-glutamine. Cellular expression of ASS by western blot was assessed using an anti-ASS antibody (Clone 25, BD Biosciences, San Jose, CA, USA). LC3B (Cell Signaling, Danvers, MA, USA), active caspase-3 (Cell Signaling), total caspase-3 (Invitrogen Life Technologies, Carlsbad, CA, USA) and actin (GeneTex, Irvine, CA, USA) were used according to the manufacturer's instructions. Topotecan hydrochloride was obtained from Axxora (San Diego, CA, USA) and chloroquine was obtained from Sigma-Aldrich (St Louis, MO, USA).

### Immunohistochemistry

Tumours and normal tissues were stained using anti-ASS antibody 195-21-1 (LICR, New York, NY, USA), as detailed in Jungbluth *et al* (2010).

### Western blot analysis

Whole-cell lysates of SCLC cell lines were prepared in RIPA lysis buffer (Tris-HCl 50 mM, 0.05% SDS, 0.5% Na-deoxycholate, NaCl 150 mM, EDTA 5 mM plus protease inhibitor cocktail buffer (Roche Applied Science, Indianapolis, IN, USA). Protein amounts were determined using the Pierce BCA protein assay (Thermo Fisher Scientific, Rockford, IL, USA) and equal amounts of proteins were resolved by SDS–PAGE using 4–12% gels (NuPAGE, Invitrogen Life Technologies). Proteins were transferred to PVDF membranes (Millipore, Billerica, MA, USA), blocked with 5% BSA and probed with appropriate antibodies overnight at 4°C. Following washing, the membranes were then probed with the appropriate secondary antibody before proteins were finally visualized using ECL reagent (Perkin-Elmer, Fremont CA, USA).

### Quantitative-real time PCR

For RNA extraction, cell pellets were dissolved in 600 *μ*l TRI Reagent solution (Ambion, Austin, TX, USA), and 60 *μ*l bromochloropane was then added. Approximately two drops of optimum cutting temperature compound (Miles Inc., Elkhart, IN, USA) was then added to the tube and the mixture was vortexed and left to stand at RT for 2 min. After centrifugation at 14 000 r.p.m. for 10 min, the supernatant was removed to a new tube where an equal volume of 100% isopropanol was added to precipitate the RNA. Following centrifugation at 14 000 r.p.m., the RNA pellet was washed in 75% ethanol and again centrifuged before re-suspension in 50 *μ*l warm water. The RNA concentration was determined using a nanophotometer (Implen Inc., Westlake Village, CA, USA).

For amplification of cDNA, 1.5 *μ*g RNA was added to a cDNA reaction mixture comprising 10 × reaction buffer (Qiagen, Valencia, CA, USA), 5 mM dNTPase (Qiagen), Oligo DT (Qiagen), reverse transcriptase (Invitrogen Life Technologies) and RNAse Out (Invitrogen) in a total volume of 20 *μ*l. For quantitative-real time (qRT)-PCR reactions, 1.5 *μ*l cDNA was mixed with a reaction mix containing 5 *μ*l SYBRGreen (Invitrogen), 0.02 *μ*l Rox, 0.2 *μ*l primers, and water for a total reaction volume of 10 *μ*l. For ASS, primers were F: 5′-TTTAAGCAGACTAAGGGG-3′ and R: 5′-CCATCCCAGGTTATAAGCACA-3′. The qRT-PCR analysis was performed using a 7500 Fast Real-Time PCR system (Applied Biosystems, Carlsbad, CA, USA), with GAPDH used for normalization of expression. Relative quantification of gene expression (relative amount of target RNA) was determined using the equation 2^(−ΔΔCT)^.

### Proliferation assays

To assess the anti-proliferative effect of ADI-PEG20 on adherent cells, cells were plated a density of 2 × 10^3^ cells per well in a tissue culture 96-microwell plate and allowed to adhere overnight. The following day, cells were treated with ADI-PEG20 (DesigneRx Pharmaceuticals, Vacaville, CA, USA, a subsidiary of Polaris Group, San Diego, CA, USA) ranging from 0 to 10 mIU ml^−1^. After incubation for 120 h, cell viability was determined using (3-(4,5-dimethylthiazol-2-yl)-5-(3-carboxymethoxyphenyl)-2-(4-sulfophenyl)-2H-tetrazolium) (MTS, CellTiter 96 AQ_ueous_ One Solution, Promega, Madison, WI, USA), according to the manufacturers protocol. Twenty microliters of MTS reagent was added to the appropriate control and assay wells before the plate was incubated for 2 h at 37°C and absorbance read at 490 nm. Absolute absorbance for each treatment was determined by subtracting the background MTS absorbance, and the mean and standard deviation was calculated. The effect of the autophagy inhibitor chloroquine (Sigma) on cell proliferation was assessed using the MTS assay.

To assess the activity of ADI-PEG20 in non-adherent cells, cells were plated at a density of 1 × 10^5^ cells per well in a tissue culture 24-well plate. Additional media was then added containing ADI-PEG20 for final concentrations ranging from 0–10 mIU ml^−1^. To measure changes in proliferation following incubation with ADI-PEG20 for 120 h, cells were collected, washed and lysed in RIPA buffer. Total protein for each treatment was then determined using the BSA protein assay as a measure of total cell number. All treatments were performed at least in triplicate.

### Propidium iodide staining for sub-G_1_ staining

Apoptosis was measured by FACS of propidium-iodide-stained cells as detailed by Riccardi *et al* (Riccardi and Nicoletti, 2006). Cells were plated in 24-well plates and treated with ADI-PEG20 for 120 h. Cells were then harvested, washed and fixed in 70% ethanol. DNA was then stained using 20 *μ*g ml^−1^ propidium iodide containing 10 *μ*g ml^−1^ DNAse-free RNAse A (Sigma-Aldrich). Cells were then read on a BD FACSCalibur (BD Biosciences) and analyzed using Flow Jo Software (Tree Star, Ashland, OR, USA).

### Small interfering RNA downregulation of ASS

To further assess the importance of cellular ASS expression in response to treatment with ADI-PEG20, expression of ASS was silenced through the use of ASS-specific siRNA. Small interfering RNA constructs were obtained from Integrated DNA technologies (IDT, Coralville, IA, USA) against the ASS-coding region. Only siRNA constructs without any other transcript matches were selected for further experiments.

Argininosuccinate synthetase-positive SW1222 were plated out at a density of 6 × 10^5^ cells per dish in 8 ml media in 100 mm tissue culture dishes and allowed to adhere overnight. For transfection, 10 *μ*l of 10 *μ*M ASS siRNA was added to 990 *μ*l Opti-MEM media and 20 *μ*l Lipofectamine 2000 reagent was diluted in 980 *μ*l Opti-MEM media (Invitrogen Life Technologies). These mixtures were incubated for 5 min at RT before being mixed and incubated for a further 20 min at RT. The transfection mixture was then added to the cells and incubated for 24 h at 37°C. At this time, preparations of the transfected cells were lifted from the culture dish, and plated out in 96-well plates in order to assess the effect of ADI-PEG20 on the growth of the transfected cells. Additional cells incubated for a further 72 h before processing for PCR and western blot analysis.

### Arginine deiminase-PEG20 *in vivo* efficacy study

The ASS-negative SCLC SK-LC-13 was found to be tumourigenic and was subsequently used to determine the efficacy of ADI-PEG20 *in vivo*. The activity of ADI-PEG20 was also assessed in mice bearing ASS-positive NCI-H69 SCLC xenografts. Small cell lung cancer xenografts were established in female BALB/c-nude mice, 3–4 weeks of age weighing ∼20 g (Charles River Labs, Wilmington, MA, USA). To establish the tumours, 10 × 10^6^ cells in media were mixed 1 : 1 with Matrigel High Concentration (BD Biosciences) and injected subcutaneously in the abdominal area of the mice. Tumour growth was regularly measured and tumour volume calculated using the formula (TV=(length × width^2^)/2). All animal studies were approved by the MSKCC Institutional Animal Care and Use Committee. Mice were euthanized when tumours reached an approximate volume of 1000 mm^3^.

The anti-tumour efficacy of ADI-PEG20 was simultaneously assessed in mice bearing either moderate or large SK-LC-13 SCLC xenografts. In the first study, treatment was initiated once tumours had reached an average size of 125 mm^3^. In the large xenograft study, treatment was initiated once tumours had reached an average size of 500 mm^3^. Arginine deiminase-PEG20 was administered at doses the of 1, 2 and 5 IU per animal once every 5 days for 20 days (five doses). To assess the effect of sustained dosing, further groups (*n*=5) at all dose levels received continued administration of ADI-PEG20 every 5 days until tumours progressed to the 1000 mm^3^ size limit. Additionally, the efficacy of ADI-PEG20 was assessed in mice bearing ASS-positive NCI-H69 xenografts. Here, mice received five doses of 2 IU ADI-PEG20 for 20 days once tumour had reached an average size of 150 mm^3^. Arginine deiminase-PEG20 was administered by intraperitoneal injection in all studies. The specific activity of ADI-PEG20 used in these studies is 9 IU mg^−1^ of protein. Thus, 1 IU of ADI-PEG20 per 20 g mouse is equivalent to 160 IU m^−2^.

### Measurement of serum arginine and citrulline

In order to determine the effect of ADI-PPEG20 treatment on systemic arginine levels, mice sera were analyzed using high-performance liquid chromatography (HPLC). L-arginine and L-citrulline were resolved with a Pickering Laboratories PCX 5200 post-column derivatization instrument (Pickering Laboratories, Mountain View, CA, USA) at 39°C reaction temperature and a fluorescence detector. All reagents, including the buffer and column, were used as suggested by Pickering Laboratories. Total ADI-PEG20 levels were measured by ELISA, as described previously (Holtsberg *et al*, 2002).

### Statistical analysis

Efficacy of ADI-PEG20 treatment *in vivo* was assessed by comparing means of control and treatment groups using unpaired two-tailed *t*-tests at the termination of control groups using GraphPad Prism (Version 5.0, GraphPad Software Inc., La Jolla, CA, USA). A 95% confidence level was used, with mean tumour volume declared significantly different if *P*<0.05.

## Results

### SCLC frequently lack expression of ASS

**Figure 1:**
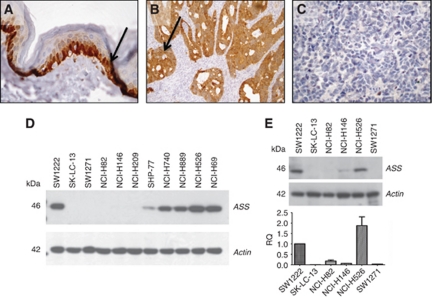
Small cell lung cancer human tumours and cell lines frequently lack expression of ASS: ASS protein expression (brown color, arrows) in normal skin (**A**), colon carcinoma (**B**) and small cell lung cancer (**C**) at high power magnification, identified using anti-ASS antibody 195-21-1 (LICR). Expression of ASS protein was assessed by western blot in a panel of SCLC cell lines and compared with positive control SW1222 colon cancer cells (**D**). Argininosuccinate synthetase protein expression in cell lines was observed to generally correlate with mRNA expression as determined by qRT-PCR (**E**).

As the lack of ASS expression is generally associated with sensitivity to ADI, its expression was assessed in human SCLC tumours. As shown in Figure 1C, an initial immunohistochemistry (IHC) analysis of human SCLC tumours revealed that some SCLC had a near total lack of ASS expression. Approximately 45% (7 out of 16) of tumours in this initial analysis demonstrated little or no ASS expression. On the contrary, robust expression of ASS was apparent in normal tissues such as skin (Figure 1A) and other cancers such as colon carcinoma (Figure 1B; Jungbluth *et al*, 2010).

As the initial immunohistochemical analysis has shown a frequent loss of ASS expression in SCLC human tumours, we have assessed the expression status of ASS in a panel of SCLC cell lines. Western blot analysis revealed that 5 out of 10 (50%) of the tested SCLC cell lines lacked significant expression of ASS on the protein level (Figure 1D). The cellular expression of ASS as detected by western immunblotting was similar using both the Clone 25 and 195-21-1 anti-ASS antibodies (data not shown). Analysis of mRNA levels using qRT-PCR demonstrated a general correlation between ASS mRNA and protein expression levels (Figure 1E).

### Arginine deiminase-PEG20 inhibits the proliferation of ASS-negative SCLC cell lines *in vitro*

**Figure 2:**
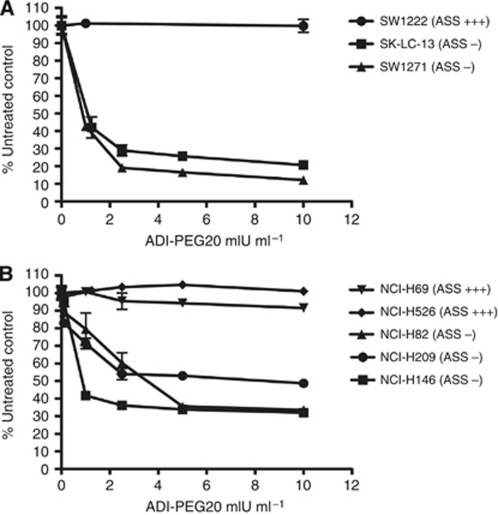
Arginine deiminase-PEG20 inhibits the *in vitro* proliferation of ASS-negative SCLC cells. Adherent (**A**) and non-adherent (**B**) cells were treated with ADI-PEG20 for 120 h before proliferation was assayed using the MTS assay for adherent cells or the BCA total protein assay for non-adherent cells.

The effect of ADI-PEG20 on cell proliferation *in vitro* was assessed in both ASS-positive SCLC cells and those lacking expression of the enzyme. We included cells with both adherent and non-adherent growing tissue culture characteristics in these experiments. We found a clear dose-dependent decrease in proliferation in the adherent ASS-negative SCLC cell lines SK-LC-13 and SW1271. No effect is apparent on the growth of the adherent ASS-positive colon carcinoma cell line SW1222 (Figure 2A). As for non-adherent cells, ASS-positive cells demonstrated almost total resistance to the anti-proliferative effects of ADI-PEG20, whereas a relative decrease in proliferation was again observed in ASS-deficient cells following ADI-PEG20 treatment (Figure 2B). As the ADI-PEG20-sensitive cell line SK-LC-13 was determined to be tumourigenic, it was chosen to be a model cell line for later experiments *in vitro* and *in vivo*.

### Arginine deiminase-PEG20 induces autophagy and caspase-independent apoptosis

**Figure 3:**
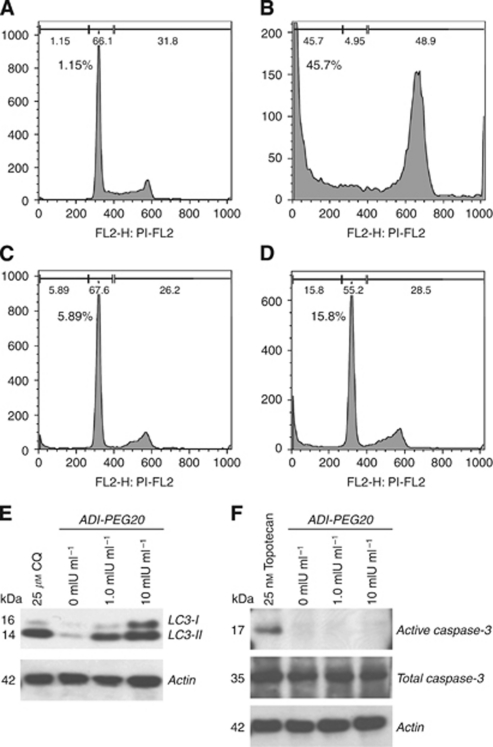
Arginine deiminase induces apoptosis and autophagy in ASS-negative SK-LC-13 SCLC cells. Fluorescence-activated cell sorting analysis of sub-G_1_ DNA content demonstrating apoptosis: cells were incubated in control media (**A**), 25 nM topotecan (**B**), 1.0 mIU ml^−1^ ADI-PEG20 (**C**) and 10 mIU ml^−1^ ADI-PEG20 (**D**) for 72 h before DNA staining with PI. An increase in LC3-II protein level following 24 h incubation with ADI-PEG20 or chloroquine (CQ)-positive control suggests autophagy (**E**). The apoptosis induced by ADI-PEG20 seems to be caspase independent, as only topotecan chemotherapy was observed to induce activation of caspase-3 in SK-LC-13 cells (**F**).

As with more general nutrient starvation, the depletion of intracellular arginine by ADI has been observed to induce metabolic stress and subsequent cellular autophagy (Kim *et al*, 2009b; Savaraj *et al*, 2010). Subsequently, we assessed if treatment with ADI-PEG20 was able to induce cellular autophagy in SCLC by assaying for the formation of the autophagy-related protein LC3-II. Although some basal expression of LC3-II expression was observed in SK-LC-13, treatment with ADI-PEG20 resulted in a clear increase in the detectable cellular level of the protein. (Figure 3E). Chloroquine is a known inhibitor of autophagy that disrupts normal lysosomal functions, and thus results in an increase in the cellular level of LC3-II. Subsequently, cells treated with chloroquine as a positive control demonstrated very robust expression of LC3-II. Combined treatment of cells with ADI-PEG20 and chloroquine resulted in a small but significant (*P*=0.008) decrease in viability relative to individual treatments, suggesting that inhibition of autophagy may enhance the efficacy of ADI-PEG20 (Supplementary Figure 1).

In order to determine the possible mechanism of the anti-proliferative effects of ADI-PEG20 a FACS analysis of apoptosis by sub-G1 DNA content was performed. Cells were treated for 72 h before the analysis was performed. Little apoptosis was detectable in untreated cells, while 25 nM topotecan, used as a positive control, was observed to cause apoptosis in around 45% of the SK-LC-13 cells (Figures 3A and B). Although not as effective as topotecan, incubation with 1.0 and 10 mIU ml^−1^ ADI-PEG20 resulted in the induction of apoptosis in ∼6% and 16% of cells, respectively (Figures 3C and D). Although apoptosis was apparent by sub-G1 DNA content, no activation of caspases were observed following treatment of cells with ADI-PEG20 in contrast to topotecan-treated cells (Figure 3F).

### Silencing of ASS expression induces sensitivity to ADI-PEG20

**Figure 4:**
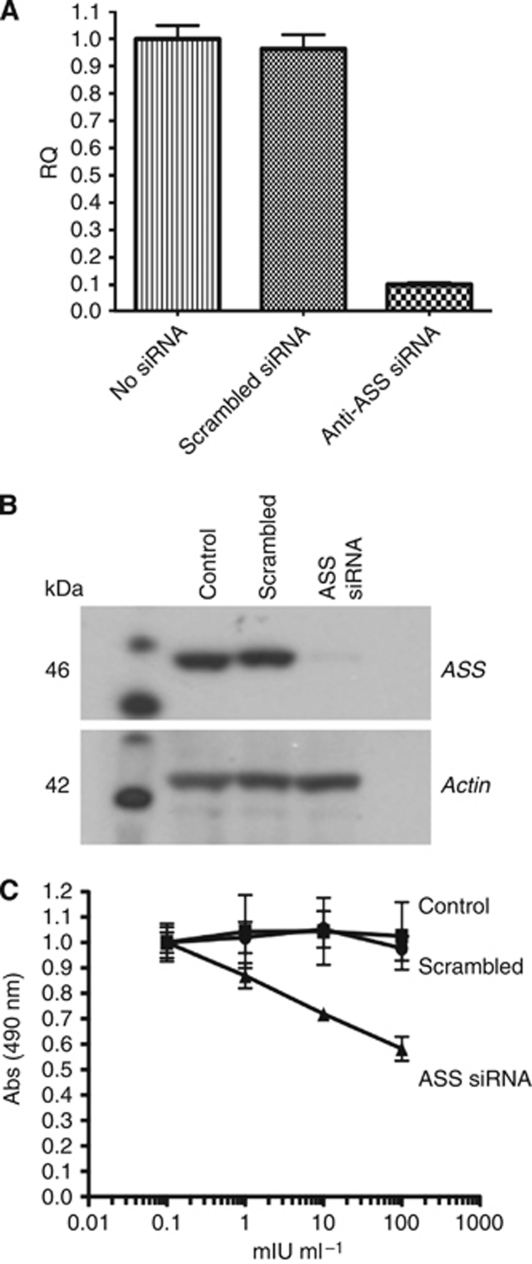
Silencing of ASS expression with siRNA. Relative expression of ASS mRNA expression determined by RT–PCR in SW1222 cells treated with ASS siRNA (**A**). Relative expression of ASS protein assessed by western blot in SW1222 cells treated with ASS siRNA (**B**). The MTS proliferation assay of ADI-PEG20-treated cells demonstrating susceptibility to anti-proliferative effects following loss of ASS expression (**C**).

Following transfection with ASS-specific siRNA, the relative expression of ASS in ASS-positive cell lines was assessed with Real Time PCR. As shown in Figure 4A, transfection with ASS siRNA reduced ASS mRNA levels by ∼90% after 72 h incubation. Simultaneous western Blot analysis demonstrated a robust reduction in the expression of ASS protein levels in cells treated with ASS-specific siRNA (Figure 4B). However, some expression of ASS remained under these conditions. Examination of cell viability using the MTS assay demonstrated that ASS-positive cells treated with ASS siRNA became sensitive to ADI-PEG20-induced arginine deprivation, resulting in reduced cell viability, whereas no decrease in viability was observed in control or scrambled siRNA-treated cells (Figure 4C).

### Arginine deiminase-PEG20 inhibits the growth of SCLC xenografts

**Figure 5:**
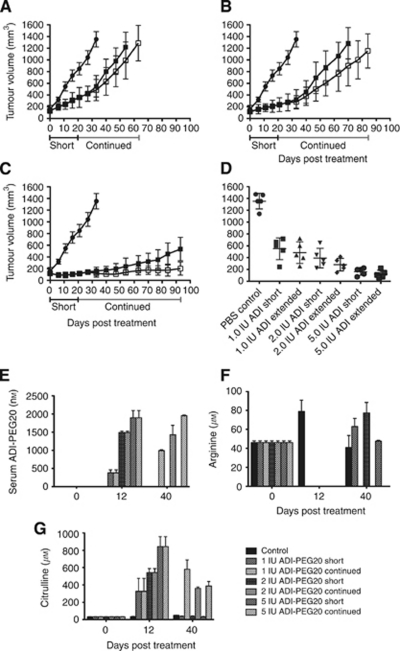
Arginine deiminase-PEG20 inhibits the growth of moderately sized SK-LC-13 SCLC xenografts in BALB/c-nude mice. Growth curves of mice receiving PBS vehicle (•), a short 20 day course (dosing every 5 days) of ADI-PEG20 (▪), or continued dosing (every 5 days until group termination) of ADI-PEG20 (□) at doses of 1 IU per mouse (**A**), 2 IU per mouse (**B**) and 5 IU per mouse (**C**) as indicated. Tumour volumes at termination of the control group on day 33 are shown in (**D**). Serum levels of ADI-PEG20 (**E**), arginine (**F**) and citrulline (**G**) are shown for days 0, 12 and 40 of the study. Values are the same in short and extended dosing cohorts at day 0 and 12, as extended dosing was only initiated at day 20.

The anti-tumor efficacy of systemic treatment with ADI-PEG20 *in vivo* was assessed in BALB/c-nude mice bearing human SCLC xenografts. Separate studies were performed in mice with established tumours around 125 mm^3^, and in mice bearing larger (∼500 mm^3^) tumours at the start of treatment. In mice bearing moderately sized SK-LC-13 xenografts (124.6±37.1 mm^3^), ADI-PEG20 caused a significant and dose-dependent reduction in tumour growth relative to control mice (Figures 5A–C). Control mice were euthanized at day 33 due to excessive tumour volume, at that time the mean tumour volume of control mice was significantly larger than those in the ADI-PEG20-treated mice groups, regardless of the dose or treatment schedule (*P*>0.0001 for all treatment groups; Figure 5D). At completion of the study, tumours treated with continued dosing of 5 IU ADI-PEG20 every 5 days were significantly smaller than those dosed every 5 days for only 20 days (*P*=0.0063). However, this effect was not observed at the 1 and 2 IU dose levels, as tumour growth proceeded at a comparable rate in groups receiving short and continued dosing. Subsequently, the mean tumour volumes of mice receiving short or continued ADI-PEG20 dosing were not significantly different at the termination of respective short dosing groups due to excessive tumour volume (1 IU: *P*=0.251; 2 IU: *P*=0.084). Treatment of ASS-positive NCI-H69 SCLC xenografts with ADI-PEG20 did not produce any effect on tumour growth (Supplementary Figure 2).

Analysis of serum from these mice before (day 0), during (day 12) and after (day 40) initial dosing of ADI-PEG20 reveals that ADI-PEG20 serum levels were dose dependent (Figure 5E). In mice where ADI-PEG20 was only administered up to day 20, serum levels of the enzyme were observed to return to baseline levels by day 40, consistent with the ∼7 day half-life of ADI-PEG20 in the mice (Ensor *et al*, 2002; Holtsberg *et al*, 2002). Further, this short course of treatment only temporarily depleted serum arginine, as expected, which subsequently returned to normal levels 20 days after the last dose (day 40) without further dosing of ADI-PEG20 (Figure 5F). Citrulline levels rose in a dose-dependent relationship to arginine, with a shorter course of ADI-PEG20 correlating with a return of citrulline levels to baseline (Figure 5G). Citrulline levels increased with extended dosing at the 1 IU level, consistent with systemic arginine remaining and being metabolized to citrulline, despite arginine serum levels being below the limits of detection. Little increase in citrulline levels was observed with continued dosing at the 2 and 5 IU dose levels.

**Figure 6:**
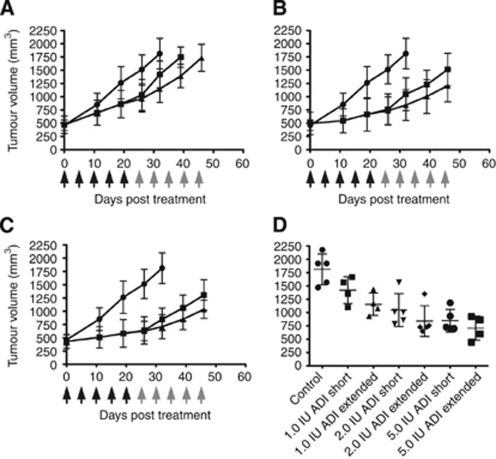
Arginine deiminase-PEG20 inhibits the growth of large SK-LC-13 SCLC xenografts in BALB/c-nude mice. Growth curves of mice receiving PBS vehicle (•), a short 20 day course of ADI-PEG20 (black arrows) (▪), or continued dosing of ADI-PEG20 (grey arrows) (▴) at doses of 1 IU per mouse (**A**), 2 IU per mouse (**B**) and 5 IU per mouse (**C**). Tumour volumes at termination of the control group on day 32 are shown in (**D**).

In the second study of ADI-PEG20 *in vivo*, treatment was begun when tumours had grown to a relatively large size of 473.4±161.0 mm^3^. A dose-dependent inhibition of tumour growth was again observed (Figures 6A–C), although this was not as significant as that observed in animals bearing smaller xenografts. Tumour volumes where compared at termination of the control cohort on day 32 of this study (Figure 6D). Continued treatment with 1 IU ADI-PEG20 was able to significantly reduce the tumour volume relative to control mice (*P*=0.007), while a short-course treatment did not result in a statistical significant reduction on tumour size (*P*=0.07). Further, continued dosing was observed to cause a moderately significant (*P*=0.26) reduction in tumour volume relative to the short-course treatment, as assessed at termination of the short-treatment group at day 39. At higher doses of ADI-PEG20, significant reduction of tumour volume relative to untreated controls was observed with both short (*P*=0.004) and continued (*P*=0.0007) administration at the 2 IU level, and also in both the short (*P*=0.0003) and continued (*P*=0.0001) schedules at the 5 IU dose level. However, continued dosing did not significantly improve responses at these doses relative to short-course treatment.

## Discussion

This study describes for the first time that a large proportion of SCLCs lack the expression of ASS, and that ASS-negative SCLC are sensitive to arginine deprivation therapy. Although patients with SCLC often demonstrate a robust initial response to chemotherapy, relapse rates remain high, highlighting the need for the development of novel therapeutic options in this disease. Argininosuccinate synthetase deficiency in SCLC was first identified in an initial study using IHC, where ∼50% of human tumours examined were found to lack expression of the enzyme (Jungbluth *et al*, 2010). A similar ratio of ASS deficiency was observed in a subsequent analysis of a panel of 10 available SCLC cell lines, with 5 cell lines demonstrating little to no ASS expression and 5 others demonstrating moderate to high levels of enzyme expression. Although the frequency of ASS deficiency does not equal the almost total absence of expression as reported in melanoma, it remains that 50% of the nearly 30 000 new cases of SCLC reported in the United States each year may be susceptible to arginine deprivation therapy.

Establishing preferential sensitivity to ADI-PEG20 in ASS-deficient SCLC tumour cells was an essential component in assessing the efficacy of ADI-PEG20 in SCLC. *In vitro* studies using both adherent and non-adherent ASS-deficient SCLC cell lines demonstrated that ADI-PEG20 caused dose-dependent anti-proliferative efficacy. These results support previous reports that have identified differential sensitivity to ADI in diverse cancers such as leukaemia and pancreatic cancer based upon the endogenous or inducible levels of ASS (Shen *et al*, 2003; Dillon *et al*, 2004; Noh *et al*, 2004; Yoon *et al*, 2007; Bowles *et al*, 2008). In SW1222 cells that express high levels of ASS, sensitivity to arginine deprivation by ADI-PEG20 could be induced by the reduction of ASS expression by ASS-specific siRNA. This sensitivity was not observed in cells transfected with control siRNA that retained high ASS expression. These results indicate that loss of ASS protein is associated with the anti-proliferative effects of ADI-PEG20 in otherwise identical cells and further validates the relative sensitivities observed in SCLC cancers of differing ASS expression.

Treatment of SCLC with ADI-PEG20, and the resultant depletion of arginine, was observed to induce cellular autophagy, as evidenced by the detection of the autophagic marker LC3-II. The induction of autophagy as measured by the formation of LC3-II appears to be a relatively ubiquitous response to ADI-PEG20 treatment in cells lines, and has been observed in cells that seem to progress to both caspase-dependent and -independent apoptosis (Delage *et al*, 2010; Savaraj *et al*, 2010). Although the induction of autophagy is associated with the mechanism of action of some cancer therapeutics, it appears that autophagy is an early protective response to cellular arginine deprivation in ADI-PEG20-treated cells (Kondo *et al*, 2005; Savaraj *et al*, 2010). A similar induction of cyto-protective autophagy has been observed in response to general amino-acid deprivation (Kondo *et al*, 2005; Steiger-Barraissoul and Rami, 2009; Savaraj *et al*, 2010). As inhibition of cyto-protective autophagy may potentiate the anti-proliferative effects of ADI-PEG20, the combination of ADI-PEG20 with 25 *μ*M of the autophagy inhibitor chloroquine was assessed *in vitro* (Kim *et al*, 2009b). However, the combination only induced a moderate, albeit significant (*P*=0.008), increase in the anti-proliferative effect of ADI-PEG20 in SCLC cells (Supplementary Figure 1).

Treatment of SCLC with ADI-PEG20 caused a moderate increase in the population of cells in sub-G_1_ peak following staining with propidium iodide, suggesting that these cells had undergone apoptosis, as had been observed in the treatment of ASS-negative leukaemia, retinoblastoma and prostate cancer (Gong *et al*, 1999, 2000; Noh *et al*, 2004; Kim *et al*, 2007, 2009b; Bowles *et al*, 2008). Western blot analysis revealed that ADI-PEG20 did not cause caspase activation in SK-LC-13 SCLC cells. These results are similar to those observed by Kim *et al* (2009a, 2009b), who found that ADI-PEG20 caused caspase-independent apoptosis in prostate cancer cell lines. In contrast, Bowles *et al* (2008) observed caspase-dependent apoptosis following treatment of pancreatic cancer cells with ADI, suggesting that different tumour types may respond differently to the effects of arginine deprivation. Similarly, restriction of methionine has been observed to induce caspase-dependent apoptosis in HeLa cells, while it induced caspase-independent death in PC-3 prostate cancer cells (Lu *et al*, 2003). The overall cellular response to arginine deprivation induced by ADI-PEG20 in SCLC cells appears to operate through a complex mechanism involving an initial metabolic response seen in the induction of autophagy, followed by caspase-independent cell death. The precise cellular pathways responsible for ADI-PEG20-induced cell death seem to be variable, and have not yet been fully elucidated (Bursch *et al*, 2008; Kim *et al*, 2009a; Savaraj *et al*, 2010).

*In vivo* studies in mice revealed that growth of SK-LC-13 xenografts was abrogated by ADI-PEG20 in a dose-dependent manner. Significant anti-tumor activity was observed at the 1, 2 and 5 IU dose per animal doses. Importantly, although more robust inhibition of tumour growth was observed in mice bearing smaller (∼120 mm^3^) established tumours, ADI-PEG20 also demonstrated significant anti-tumor efficacy in mice bearing large (∼500 mm^3^) xenografts. Although direct comparison with other tumour types is difficult, the anti-tumor efficacy observed with ADI-PEG20 in SCLC xenografts is similar and possibly superior to that observed using equivalent doses of ADI-PEG20 in different tumour types including renal, pancreatic and prostate cancer xenografts. Although continued treatment with ADI-PEG20 did not induce more significant tumour inhibition at all of doses investigated, the efficacy observed with long-term administration of 5 IU ADI-PEG20 suggests that continued treatment of ADI-PEG20 may be advantageous in the clinical assessment of ADI in SCLC. As the *in vivo* data suggests ADI-PEG20 may often lead to tumour stabilization rather than an absolute reduction in tumour volume, it is likely that assessment of clinical efficacy will need to include measures such as overall survival.

To date, the majority of clinical studies assessing the efficacy of ADI-PEG20 in patients with ASS-negative tumours have used doses ⩽160 IU m^−2^, as this ‘Optimal Biological Dose’ was able to reduce serum arginine <2 *μ*M (Izzo *et al*, 2004; Ascierto *et al*, 2005; Delman *et al*, 2005; Glazer *et al*, 2010). Although these doses are well tolerated and some signs of disease stabilization have been observed, they have yet to produce sustained tumour responses in these patients (Izzo *et al*, 2004; Ascierto *et al*, 2005). Analysis of serum in ADI-PEG20 treated mice in this study indicated that although lower doses are able to effectively reduce plasma arginine levels to below the limit of assay detection, levels of citrulline are observed to rise with continued dose escalation. As citrulline levels in ADI-PEG20 treated mice are directly associated with degradation of arginine to citrulline through the enzymatic activity of ADI, rising citrulline levels are indicative of continued breakdown of systemic arginine. Subsequently, it is apparent that reduction in plasma arginine levels to very low levels does not signify that biochemical neutrality of systemic arginine had been reached. Therefore, further dose escalation may be required for the total removal of systemic arginine that is considered necessary for the greatest efficacy of arginine deprivation therapies such as ADI (Feun *et al*, 2010).

In spite of the likely benefits of aggressive dosing with ADI, impediments to higher doses in patients have been observed in the clinical assessment of ADI-PEG20 due to limitations of the volume administered via the intramuscular injection route used. Subsequently, injections at multiple sites or alternative routes may need to be investigated for greater dose escalation. Additionally, although pegylation dramatically reduced the immunogenicity of ADI, the potential for robust immunological responses against the enzyme remains a concern in the clinic and may limit the course of ADI-PEG20 administration. Some evidence of rising serum arginine and anti-ADI antibody levels have been observed in patients receiving ADI-PEG20, although not all antibodies to the enzyme are neutralizing (Izzo *et al*, 2004; Ascierto *et al*, 2005; Ott *et al*, 2009; Delage *et al*, 2010; Yang *et al*, 2010).

Additionally, prolonged treatment with ADI-PEG20 may lead to the induction of ASS expression and the activation of other cellular pathways associated with resistance to apoptosis, possibly limiting the overall treatment window for ADI (Shen *et al*, 2003; Tsai *et al*, 2009; Kim *et al*, 2009b; Delage *et al*, 2010). Small cell lung cancer xenografts treated continuously with 5 IU ADI-PEG20 study demonstrated a sustained response to ADI-PEG20 for the 90-day period of the study, suggesting that at least at this dose, profound resistance to the anti-proliferative effect of ADI-PEG20 had not been induced. However, it is possible that in the clinical setting, ADI-PEG20 resistant tumours could arise in SCLC, as has been observed in melanoma cell lines (Tsai *et al*, 2009). Further studies will aim to assess whether any induction of ASS is observed following treatment of SCLC with ADI-PEG20, and whether resistance to ADI-PEG20 subsequently becomes apparent in the SCLC tumour models. The possibility of induced resistance again highlights the likely benefit from relatively aggressive treatment regimes and provides the rationale for combination of ADI-PEG20 with other therapies with distinct mechanisms of action. Subsequently, the combination of ADI-PEG20 with other drugs aiming to enhance the efficacy of ADI or complement it through separate cell death mechanisms is under active investigation (Noh *et al*, 2004; Delman *et al*, 2005; Feun *et al*, 2008; Kim *et al*, 2009b; Delage *et al*, 2010). The widespread absence of ASS expression in SCLC and the encouraging anti-tumor activity observed in the current study suggest that further exploration of ADI-PEG20 in the treatment of SCLC is warranted. As a result of these preclinical studies, a clinical trial of arginine deprivation therapy with ADI-PEG20 in patients with ASS-negative SCLCs has been initiated.
