# Supplementary material for: Arginine deiminase PEG20 inhibits growth of small cell lung cancers lacking expression of argininosuccinate synthetase
Source: Br J Cancer. 2011 Dec 1;106(2):324–32. doi: 10.1038/bjc.2011.524 (PMC3261683; doi:10.1038/bjc.2011.524)
Supplement: Supplementary Figures [file bjc2011524x1.doc]

SUPPLEMENTAL FIGURES

**
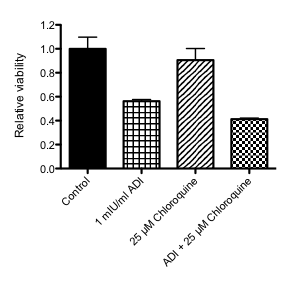
**

**Supplemental Figure 1.** Co-treatment with ADI-PEG20 and autophagy inhibitor chloroquine caused a small decrease in cell viability relative to the respective treatments.


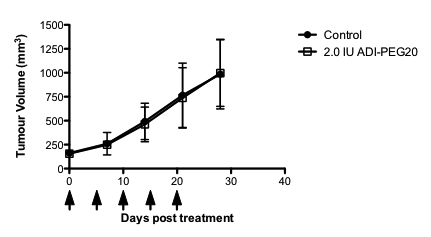


**Supplemental Figure 2.** ADI-PEG20 does not inhibit the growth of ASS positive NCI-H69 SCLC xenografts in BALB/c nude mice. Mice received PBS vehicle or 2IU/mouse

ADI-PEG20 (black arrows).
